# EEG Source Reconstruction Reveals Frontal-Parietal Dynamics of Spatial Conflict Processing

**DOI:** 10.1371/journal.pone.0057293

**Published:** 2013-02-25

**Authors:** Michael X Cohen, K. Richard Ridderinkhof

**Affiliations:** 1 Department of Psychology, University of Amsterdam, Amsterdam, the Netherlands; 2 Department of Physiology, University of Arizona, Tucson, Arizona, United States of America; 3 Cognitive Science Center Amsterdam, University of Amsterdam, Amsterdam, the Netherlands; University Medical Center Groningen UMCG, Netherlands

## Abstract

Cognitive control requires the suppression of distracting information in order to focus on task-relevant information. We applied EEG source reconstruction via time-frequency linear constrained minimum variance beamforming to help elucidate the neural mechanisms involved in spatial conflict processing. Human subjects performed a Simon task, in which conflict was induced by incongruence between spatial location and response hand. We found an early (∼200 ms post-stimulus) conflict modulation in stimulus-contralateral parietal gamma (30–50 Hz), followed by a later alpha-band (8–12 Hz) conflict modulation, suggesting an early detection of spatial conflict and inhibition of spatial location processing. Inter-regional connectivity analyses assessed via cross-frequency coupling of theta (4–8 Hz), alpha, and gamma power revealed conflict-induced shifts in cortical network interactions: Congruent trials (relative to incongruent trials) had stronger coupling between frontal theta and stimulus-contrahemifield parietal alpha/gamma power, whereas incongruent trials had increased theta coupling between medial frontal and lateral frontal regions. These findings shed new light into the large-scale network dynamics of spatial conflict processing, and how those networks are shaped by oscillatory interactions.

## Introduction

Cognitive control is crucial to goal-directed behavior, and includes our ability to adapt behavior according to conflicts, errors, or negative performance feedback [1]. One major aspect of cognitive control is inhibiting irrelevant and distracting information while focusing on task-relevant information. The medial frontal cortex (MFC) is believed to be the fulcrum of the cognitive control system, by signaling behaviorally relevant events, particularly those with negative valence such as response conflict, errors, and negative feedback [1], [2], [3]. These signals are used to recruit other prefrontal structures to facilitate implementing behavioral adjustments [4], [5], as well as directly implementing adjustments [6]. One commonly used task for studying conflict and cognitive control is the Simon task, in which subjects respond according to stimulus color while ignoring task-irrelevant spatial information. Conflict is induced when, for example, the stimulus appears in the right visual hemifield but requires a left-hand response. One advantage of the Simon task over, for example, the standard flankers task, is that the Simon task is amenable to anatomically specific hypotheses, because the conflict-producing dimension (spatial location) can be localized with noninvasive techniques like EEG or MEG. The objective of this experiment was to use EEG recordings and source space reconstruction to localize and characterize the network dynamics of cognitive control during spatial conflict processing.

Neural computations in the MFC, and its interactions with other cortical and subcortical areas, seem to be coordinated by electrophysiological oscillations in the theta (4–8 Hz) band [7], [8], [9], [10], [11], [12], [13], [14], [15]. Our and others’ recent findings suggest that MFC acts as a “hub” for large-scale network formation and information integration [16], and that the theta band is the substrate of functional communication between MFC and task-relevant areas of lateral prefrontal cortex [7], [13], occipital cortex [17], [18], motor cortex [19], and the ventral striatum [20].

On the other hand, posterior parietal regions have also been implicated in spatial conflict processing. FMRI studies show parietal activation during spatial conflict tasks [21], [22], [23], [24], and lateralized event-related potentials also confirm contralateral posterior involvement in spatial conflict [25]. Furthermore, conflict in only one visual hemifield modulates activity selectively at contralateral posterior parietal electrodes [26]. Transcranial magnetic stimulation to parietal areas attenuates the Simon effect [27], [28], maximally with stimulation around 130–160 ms after stimulus-onset [29]; similar effects are obtained when stimulating the frontal eye fields [30]. On the other hand, studies on spatial information processing implicate regions in the posterior parietal cortex (the “dorsal stream”) [31], [32], particularly in the right hemisphere [33]. These effects are often studied using event-related potentials, but spatial attention and spatial information processing are also supported by alpha-band [13], [34] and gamma-band [35], [36] oscillations.

Thus, it appears that both frontal and posterior parietal regions are implicated in conflict resolution, particularly when the conflict results from task-distracting spatial information. This leads to the hypothesis that frontal and parietal regions form a functionally coupled network during spatial conflict processing. Large-scale networks between frontal and parietal areas have been identified during working memory [37], [38], sensory decision-making [36], and cued attention tasks [39], [40]. It remains unknown how this network might facilitate spatial conflict resolution, although frontoparietal connectivity has been observed during errors [18], [41], and these networks seem to be disrupted in ADHD [42].

The purpose of the present study was to investigate how parietal cortex and MFC form a network to resolve response conflict induced by incongruence between spatial position and the required response hand. Our main hypothesis was that parietal alpha and/or gamma activity, contralateral to the stimulus presentation hemifield, would be modulated by conflict, and that connectivity between parietal alpha/gamma and frontal theta would also be modulated by conflict. We applied linear constrained minimum variance beamforming, which is a source reconstruction technique that involves designing a series of spatial filters, based on electrode positions, brain anatomy, and time-varying changes in frequency-band specific activity covariances. These spatial filters allow the estimation of time-frequency dynamics in brain space. Because beamforming has not previously been applied to EEG response conflict tasks, we also replicated conflict effects previously observed at the scalp-level (e.g., midfrontal theta conflict modulation and connectivity with lateral prefrontal regions).

## Results

### Behavioral Results

**Figure 1:**
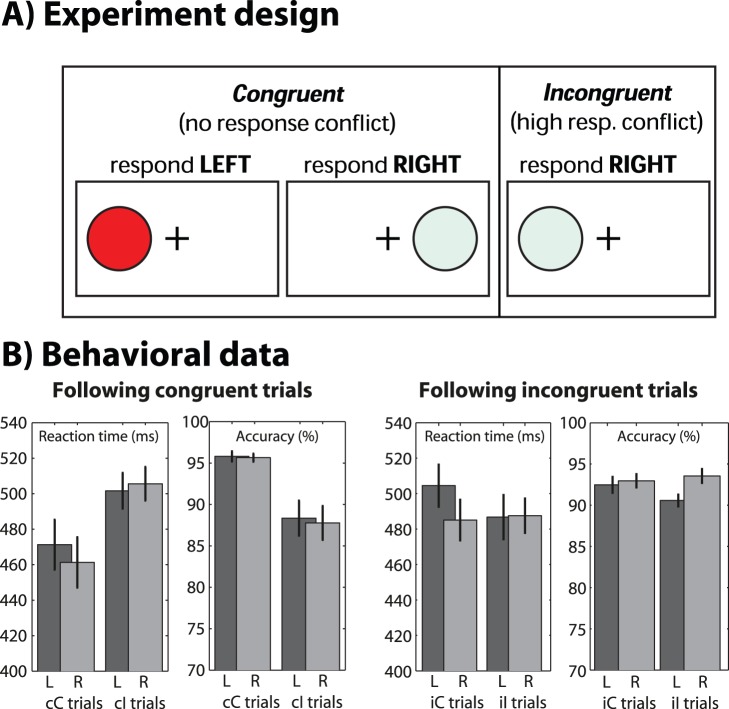
Overview of task (A) and behavioral results (B). “L” and “R” indicate trials in which the stimulus was presented on the left and right side of the screen, respectively. “cC” and “cI” indicate congruent and incongruent trials, respectively, following congruent trials.

Behavior analyses were computed using SPSS software with a repeated-measures ANOVA for “stimulus hemifield” X “condition” on reaction times (RTs) and accuracy rates, on trials following congruent trials. As expected, RTs were faster on congruent compared to incongruent trials (repeated-measures ANOVA; F_1,19_ = 44.02, p<.001). There was no main effect of stimulus hemifield (F_1,19_ = 1.89, p = .185) or interaction (F_1,19_ = 3.68, p = 0.070) (Figure 1b). Overall, behavior results showed a robust conflict effect, with a trend towards increased conflict effect for left- compared to right-hemifield stimulus trials. On trials following incongruent trials, there were no statistically significant main effects or interactions (F’s<3). The lack of behavioral manifestation of conflict following incongruent trials is consistent with known trial sequence effects in cognitive control tasks, particularly the Simon task [43].

### Conflict-related Power in Electrode-level Analyses

**Figure 2:**
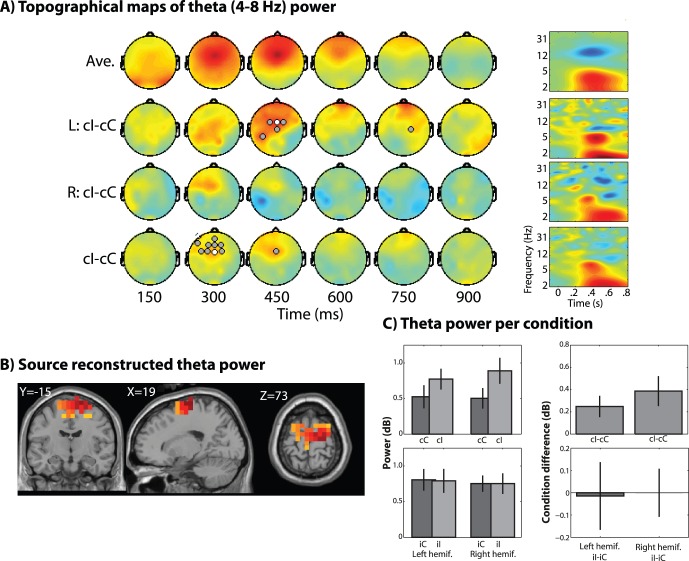
Sensor- and source-level topographical maps of task-related theta band (4–8 Hz) power. (A) Topographical maps over time (columns) in ms after stimulus onset, averaged across all conditions (top row), for incongruent-congruent trials separately for trials with left hemifield presentation (“L: I-C”) and right hemifield presentation, and for incongruent-congruent trials pooling across both left- and right-hemifield trials. Gray and white circles indicate electrodes in which the condition difference is significant at p<0.00078125 (0.05/64, thus correcting for multiple comparisons across electrodes) and p<0.005 (uncorrected), respectively. Time-frequency plots are from midfrontal electrode FCz. (B) Source-reconstructed theta power from the statistical contrast of all incongruent vs. congruent trials. X, Y, and Z correspond to MNI coordinates of displayed slices. (C) Theta power (in dB relative to pre-stimulus baseline) from all voxels in the region illustrated in panel B plotted separately for each condition and for the “conflict effect” (incongruent minus congruent trials). “hemif.” stands for “hemifield” of stimulus presentation. Top two plots show activity on trials following congruent trials; bottom two plots show activity on trials following incongruent trials. Error bars are standard errors of the mean. Data in B and C are taken from the average of 200 to 600 ms.

**Figure 3:**
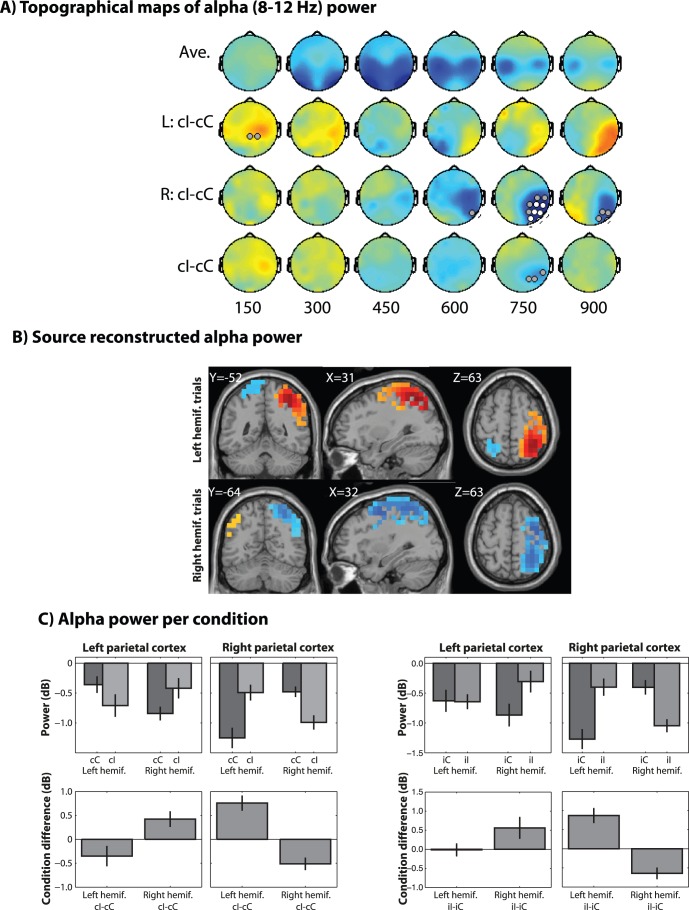
Same asFigure 2, but for conflict modulation of alpha-band (8–12 Hz) power. Bar plots in panel C show power from the contralateral hemifield activations shown in panel B (red/yellow blobs). Data in B and C are taken from the average of 600 to 900 ms.

**Figure 4:**
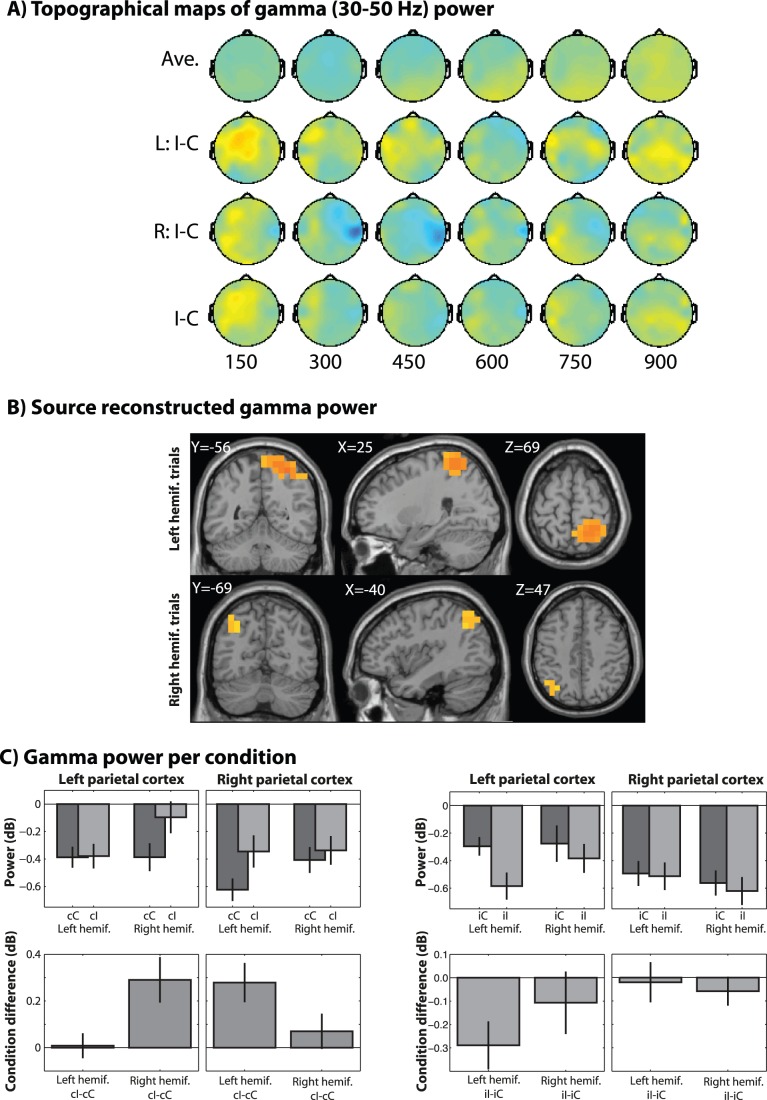
Same asFigures 2 and 3, but for gamma-band power (30–50 Hz). Data in B and C are taken from the average of 200 to 400 ms. Units on the y-axis are dB change from pre-stimulus baseline period.

We first analyzed the electrode-level data, shown in Figures 2a, 3a, and 4a. Replicating several previous studies, we found significantly increased theta power, localized to electrode FCz, for high- compared to congruent trials, around 300–450 ms post-stimulus (Figure 2a), which was statistically robust when pooling left- and right-hemifield trials. Alpha-band activity showed ipsilateral conflict-related suppression around 600 to 900 ms (Figure 3a). Finally, gamma-band (30–50 Hz) activity showed no supra-threshold results (Figure 4a).

### Conflict-related Power in Source-level Analyses

Based on the scalp-level analyses, we hypothesized that posterior MFC areas such as the dorsal anterior cingulate or pre-SMA/SMA would exhibit a theta power conflict modulation. We observed that incongruent compared to congruent trials were associated with increased theta power in posterior MFC in the pre-SMA/SMA area (see Figure 2 for MNI center coordinates). This was significant for both left- and right-hemifield trials (Figure 2b–c), and was significant from 200 to 600 ms. This conflict modulation was not observed following incongruent trials (Figure 2c, bottom row), demonstrating that that the MFC theta increase reflects online conflict processing and not other task features that are common across trials following congruent and incongruent trials.

Alpha-band activity showed significant conflict-related power that was enhanced in lateral parietal/sensorimotor areas in the stimulus hemifield-contralateral hemisphere, and suppressed in the ipsilateral hemisphere, around 600–900 ms (this is after the mean RT and thus during the inter-trial-interval) (Figure 3b–c). Note that alpha activity was in all cases was suppressed relative to the pre-stimulus baseline, and thus the “positive” activations were driven by relatively less suppression, as seen in the bar plots in Figure 3c. Interestingly, alpha activity showed a highly similar pattern of results following congruent and incongruent trials (Figure 3c). Thus, it appears that contra-lateral posterior alpha activity is related to the stimulus physical position, rather the online conflict processing per se.

Gamma-band (30–50 Hz) power showed an early conflict effect in parietal regions contralateral to stimulus presentation, beginning around 200 ms post-stimulus (Figure 4b–c). Inspection of the change in power from baseline shows that, similar to alpha, the relative increase in stimulus-contralateral gamma power for incongruent trials actually reflected relatively less suppression, compared to congruent trials. The main differences between the pattern of task-related alpha and gamma power are the lack of conflict-related suppression ipsilateral to stimulus hemifield, which is present for alpha but not for gamma, and that the alpha effect was several hundred ms after the gamma effect. Similar to the MFC theta effect, and in contrast to the posterior alpha effect, there was no significant modulation of conflict in contralateral parietal gamma following incongruent trials (left and right parietal cortex, respectively: t_19_ = −1.23 and 0.31, p = 0.23 and 0.76; Figure 4c). Thus, early parietal stimulus-contra-lateral gamma activity reflected online conflict processing and was not significantly modulated simply by the location of the stimuli.

### Connectivity with MFC Theta

**Figure 5:**
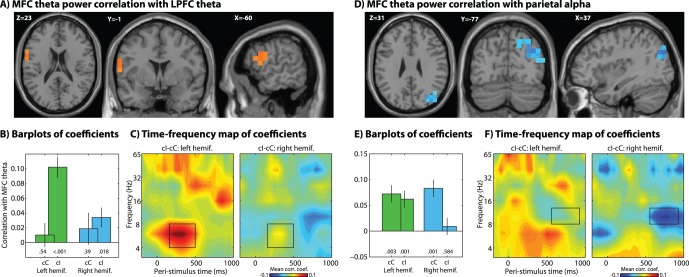
Source-space connectivity analysis seeded from the MFC region exhibiting a significant theta conflict effect (see red blob in **Figure 2b**) reveals theta-band connectivity (trial-to-trial power correlations) with left lateral prefrontal cortex. Bar plots show average correlation coefficients, with error bars reflecting standard errors of the mean, across subjects. Numbers under bars indicate p-values of correlation coefficients of each condition, tested across subjects against zero. Note that the contrast was defined as stronger correlations for incongruent compared to congruent trials, pooling across both left- and right-hemifield conditions.

**Figure 6:**
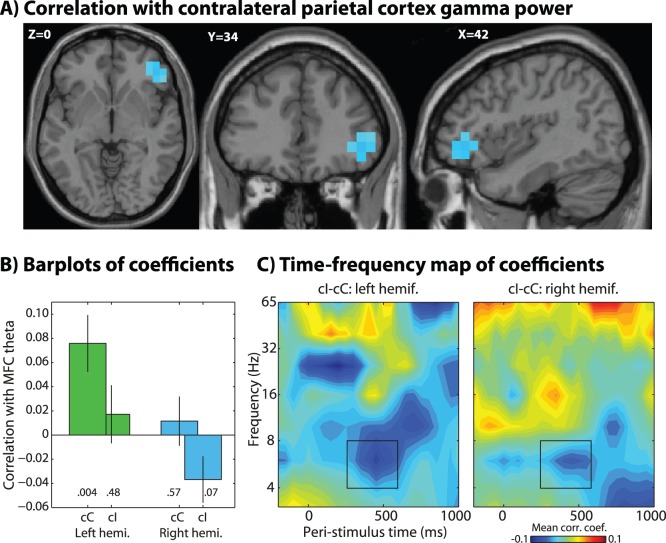
Similar toFigure 5, except the seed region was parietal gamma (see orange blobs in Figure 3b) from 200–400 ms, and the connectivity region shown here is in the theta band from 250 to 600 ms.

**Figure 7:**
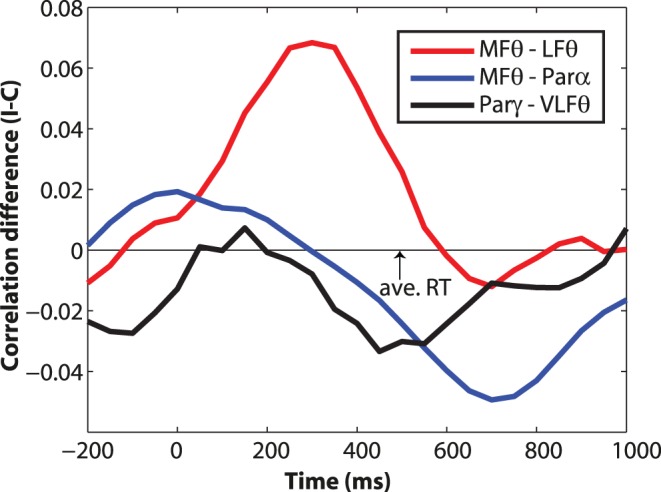
Connectivity time courses reveal dynamics of conflict-induced network shifts. Y-axis shows the difference in correlation coefficients for incongruent minus congruent trials (averaging across left- and right-hemifield trials; positive numbers indicate stronger coupling during incongruent trials, and negative numbers indicate stronger coupling during congruent trials). 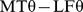 = connectivity between medial frontal theta and lateral frontal theta, see Figure 5a–c; 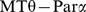 = connectivity between medial frontal theta and parietal alpha, see Figure 5d–f; 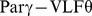 = connectivity between parietal gamma and ventrolateral frontal theta, see Figure 6.

**Figure 8:**
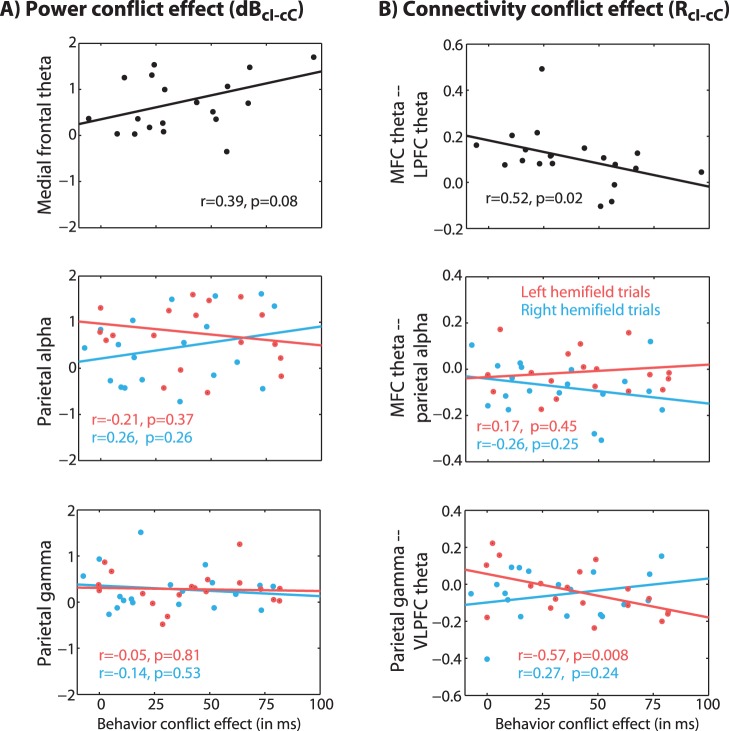
Correlations between brain conflict effects (localized band-specific power in A and network connectivity in B) and the behavior conflict effect. Brain data reflect the same time-frequency-space windows as used in previous figures; behavior conflict effect refers to individual differences in cI-cC RTs. Results show that subjects with stronger MFC-LFC theta networks, and subjects with stronger right parietal gamma-VLPFC theta networks, expressed less conflict at the behavioral level. Data are collapsed across left- and right-hemifield trials for MFC data (top row), because brain effects showed no laterality.

We performed a connectivity analysis that was designed to reveal patterns of connectivity with MFC theta over time, frequency, and space (see Methods; results are shown in Figures 5 and 6). For connectivity analyses, we focused only on trials following congruent trials. Based on previous sensor-level and dipole-modeling studies of frontal theta during conflict tasks [7], [11], [13], [19], we hypothesized that the MFC theta region identified in the power analysis (Figure 2b) would exhibit conflict-modulated connectivity with lateral prefrontal areas. Consistent with previous findings, MFC theta was significantly coupled to lateral PFC theta (in particular, in the left inferior frontal gyrus), as revealed in the contrast of incongruent vs. congruent trials (pooling over left- and right-hemifield trials) (Figure 5a; see also red line in Figure 7). Inspection of connectivity per condition in Figure 5b shows that there was significant theta-band connectivity during both left and right hemifield incongruent trials, and no significant MFC-lateral PFC connectivity during congruent trials. Time-frequency maps of correlation coefficients in Figure 5c confirm the specificity of the correlation to the theta band, particularly during left hemifield trials (as well as some trend-level coupling between MFC theta and lateral PFC gamma). Cross-subjects correlation analyses shown that those with stronger conflict-related MFC-lateral PFC networks had smaller conflict adaptation effects (p = 0.02, Figure 8b).

We next tested for connectivity in the alpha and gamma bands. Although we did not have strict hypotheses concerning exactly where and when connectivity would emerge, we generally expected to find connectivity with posterior parietal areas in the alpha and/or gamma bands, based on their conflict modulations. We observed, in contrast to the conflict-related *increase* in MFC-lateral PFC coupling, a significant *decrease* in conflict-related coupling between MFC theta and posterior parietal alpha, as seen in Figure 5d–f; see also blue line in Figure 7). Note that the negative activation is a difference between correlation coefficients; we therefore inspected the coefficients separately per condition. Bar plots in Figure 5e show that the correlation between MFC theta and parietal alpha was positive, and decreased on incongruent trials, becoming non-significant for right hemifield trials. The relative decrease in coupling occurred largely after the mean RT, which can also be seen in Figure 7. Because the seed region was MFC theta from 200 to 600 ms, this correlation can be interpreted such that MFC theta dynamics early in the trial influence parietal alpha several hundred ms later, during the inter-trial-interval. For convenience, we will refer to this network as the “alpha-related” network, to disentangle it from the network described in the next section.

It seems that incongruent compared to congruent trials are associated with a shift in network configuration from frontal-parietal (congruent trials) to MFC-lateral PFC (incongruent trials). The robustness of this shift was confirmed by a significant main effect of “network” in a hemifield X “network” (MFC-lateral PFC theta vs. MFC-parietal alpha) repeated-measures ANOVA on the difference in correlation coefficients between incongruent and congruent trials (F_1,19_ = 23.43, p<0.001). This ANOVA was performed post-hoc, inspired by the observed pattern of results.

### Connectivity with Parietal Gamma

Because parietal gamma was the earliest modulation of power by the conflict manipulation, we used these regions as seeds for the next connectivity analysis. Although we did not have strict hypotheses concerning exactly where and when connectivity would emerge, we hypothesized that stimulus-contralateral parietal gamma-seeded connectivity would be observed with frontal regions in the theta band. We found that theta power in right ventrolateral PFC exhibited a significant conflict-related change in functional connectivity with parietal gamma (Figure 6a; see also black line in Figure 7). Inspection of the correlation coefficients for each condition (Figure 6b) shows that parietal gamma-PFC theta correlations were positive for congruent trials, but non-significant or trending towards negative for incongruent trials. Left hemifield-only trials showed a more extensive PFC network including dorsolateral PFC and MFC, with conflict-related decoupling between theta and right parietal gamma power (results not shown).

Thus, the pattern of conflict-modulation of connectivity was similar as that between MFC theta and parietal alpha. Applying the same post-hoc repeated-measures ANOVA as before but substituting parietal alpha-MFC theta correlations for parietal gamma-ventrolateral PFC theta correlations also revealed a significant cross-over interaction (F_1,19_ = 12.57, p = 0.002). For convenience, we will refer to this network as the “gamma-related” network in the Discussion.

## Discussion

Here we characterized frontal-parietal networks involved in processing and resolving spatial conflict, involving MFC theta, and parietal gamma and alpha activity contralateral to the stimulus presentation hemifield. These findings reveal dynamic interactions among large-scale cortical networks during spatial conflict processing.

### Medial Frontal Cortex and Conflict Processing

The electrode-level findings displayed in Figure 2a replicate results from several previous papers implicating midfrontal topographical regions in conflict processing. Conflict modulations of MFC theta are observed across several different conflict-inducing tasks [44], [45], including conflict at the stimulus- and response-levels [19] and semantic level [13]. Further, MFC theta-band activity shows conflict- and error-related directed synchronization to occipital regions after errors [18], even those made unconsciously [17]. Here, beamforming identified the posterior medial frontal cortex as being the likely generator of this effect. While dipole fitting studies typically report the anterior cingulate cortex as the main generator [46], the present data seem to emphasize more superficial sources in the SMA/preSMA region, consistent with other distributed source imaging studies [47]. Assuming that areas in the posterior MFC (anterior cingulate cortex and the SMA/preSMA) act as a generic and high-level conflict monitoring and action selection systems during cognitive control tasks [1], [48], it is not surprising that the conflict-related activity did not distinguish between left- and right-hemifield trials. Conflict-related theta was not observed following incongruent trials, consistent with the lack of behavioral conflict effect after incongruent trials. It is believed that the cognitive control system is maximally active following incongruent trials, such that conflict can be rapidly and adequately processed; in contrast, phasic activation of conflict monitoring systems are preferentially activated after congruent trials [1], [43], [48]. Thus, the absence of a conflict modulation of MFC theta following incongruent trials helps link MFC theta to online conflict processing rather than stimulus or trial features.

### Parietal Cortex and Spatial Conflict Processing

Conflict processing/resolution are not uniquely computed via theta-band dynamics in the MFC. Rather, a network of brain regions is necessary to coordinate the identification and resolution of conflict, and MFC theta activity appears to be a hub within this network [16]. The involvement of posterior parietal areas in conflict processing has been identified in spatial conflict tasks such as a lateralized flankers task [26], although an oscillatory correlate of a role of parietal cortex in the Simon task has not yet been identified.

Thus, a novel finding in our study is the identification of an early stimulus-locked conflict modulation of parietal gamma-band (30–50 Hz) activity in the hemisphere contralateral to stimulus presentation. From the bar plots in Figures 3c and 4c, it is apparent that this gamma-band conflict-effect reflected relatively less gamma-band suppression with respect to the pre-stimulus baseline. Given that spatial information is always irrelevant in the Simon task (though it is either conflicting or facilitatory), we speculate that the pattern of results suggests that spatial processing in parietal cortex was kept under task-related inhibition, but was captured by exogenous attention during incongruent trials. One might argue that the contra-lateral gamma band effect reflected motor preparation or sensory-motor transformation rather than conflict per se. However, the lack of a stimulus-contralateral effect following incongruent trials (when subjects exhibited no behavioral manifestation of the effect of conflict) clearly links this gamma dynamic to online conflict processing rather than motor preparation or sensory-motor transformation (which would be present on all trials regardless of previous trial events).

We interpret these results to indicate that during the Simon task, parietal areas involved in spatial attention were “captured” by the stimulus (early gamma effect), and then subsequently were actively inhibited to prevent the irrelevant spatial information from affecting task performance (later alpha effect). Indeed, gamma is thought to reflect active processing [49] whereas several theories implicate alpha in goal-directed inhibition of task-irrelevant information [50], [51]. However, considering that the alpha-band effect occurred later than the gamma effect, and the lack of significant correlation between alpha-band activity and behavioral conflict, it is possible that the alpha response did not reflect a process critical to “online” conflict processing and resolution, but rather reflected a process more related to temporally extended dynamics, such as a lingering inhibition to the irrelevant and conflict-producing spatial information. Indeed, parietal alpha was the only dynamic examined here that exhibited a significant conflict effect following incongruent trials, suggesting that the posterior alpha activity reflects the spatial incongruence present on each trial, rather than online conflict resolution. Post-conflict/error alpha dynamics have been previously reported to occur between response and next trial onset [18], [52], [53], although the present findings do not fit entirely into this literature, as described below. Perhaps these interpretations could be tested more specifically in future studies in which the extent to which spatial information is relevant is specifically manipulated.

### Complementary Large-scale Cortical Networks during Congruent vs. Incongruent Conditions

We identified two types of cortical networks: One involving medial frontal and lateral prefrontal regions that operate in the theta band, and one involving stimulus-contralateral parietal cortex and areas of prefrontal cortex that exhibit cross-frequency coupling between frontal theta and parietal alpha and gamma.

The prefrontal-theta network increased in connectivity during incongruent trials, and exhibited no significant connectivity during congruent trials. This replicates previous work using single-dipole solutions [13] and Laplacian-transformed electrode data [7], [16], [54]. The present results thus contribute to this literature by confirming conflict-related enhanced theta-band connectivity using a different spatial filtering approach–beamforming–instead of phase synchronization as in previous studies. In addition, they appear to point specifically to the IFG, an area that has been implicated in action inhibition and action override [48], [55].

Of more novelty, however, is the identification of the alpha-related and gamma-related frontal-parietal network, which utilizes cross-frequency coupling (frontal theta and parietal alpha and gamma activity), and is significantly more engaged during congruent compared to incongruent trials. Long-range cross-frequency coupling has been identified as an important means of communication between brain regions [56], [57]. The connectivity between stimulus-contralateral parietal gamma and ventrolateral prefrontal theta-band activity a few hundred milliseconds later may reflect the parietal cortex providing “bottom-up” information during congruent trials (when the spatial information facilitates task processing), which is suppressed during incongruent trials (when the spatial information impairs task processing), perhaps to facilitate inhibition of the conflicting spatial location and its corresponding hand. These are speculative interpretations, and therefore should be confirmed in future studies, for example by manipulating the extent to which distracting spatial information provides a bottom-up signal (that is, the extent to which it “pops out”). Furthermore, this interaction may be specific to tasks involving salient spatial features. Other cognitive control tasks that manipulate conflict may utilize comparable mechanisms of providing feed-forward information, such as occipital cortex for visual/perceptual conflict tasks [17], [18]. It is not clear why this correlation was significantly positive only from right parietal cortex (left-hemifield stimuli). It is possible that this asymmetry is related to a right-hemisphere dominance for orienting attention [33] and early spatial conflict processing [29].

The alpha-related network was complementary in some ways to the gamma-related network: both involved interactions between parietal and frontal areas, and both were suppressed during incongruent compared to congruent trials. They diverged in other spatial and temporal aspects: whereas the gamma-related network involved early parietal activity and later PFC activity, the alpha-related network involved early MFC activity and later parietal alpha activity. Indeed, the correlation with alpha peaked at around 700 ms, over 200 ms after the mean RT and thus during the inter-trial-interval. Thus, the functional significance of this network interaction during spatial conflict resolution is unclear. Previous studies on the role of inter-trial-interval alpha and conflict/errors have suggested that this alpha power reflects a more general attention mechanism [18], [52]. Here, however, the alpha activity remained lateralized with respect to stimulus location, suggesting a more task- and trial-specific lingering suppression of the irrelevant stimulus hemifield. The findings also do not readily fit into currently popular theories of alpha: There is no visual perception during maximal alpha power/connectivity, so ideas of phase-pulsed stimulus processing [50], [51], attentional selection [50], memory selection/maintenance [58], or temporal expectation [59] do not seem immediately relevant (note that the inter-trial interval was jittered and the trial-to-trial mapping of stimulus-hemifield was balanced across trials).

### Advantages of EEG Beamforming for Studying Conflict Processing

There are three advantages of applying adaptive frequency band-specific beamformers to cognitive scalp electrophysiology data. The first is that the adaptive spatial filter increases sensitivity for resolving activity that might be otherwise difficult to measure at the electrode level. For example, gamma-band activity might not show supra-threshold results due to decreased signal-to-noise, but to the extent that this activity has a reliable spatial configuration, spatial filters such as beamformers are more likely to recover these patterns of activity, even with only 64 electrodes (typical MEG beamforming applications use 200–300 sensors).

A second advantage of beamformers is that they are designed to reconstruct both the phase-locked and non-phase-locked activity. This is because the weights are constructed in part from time-varying, frequency band-specific, electrode-by-electrode covariance matrices. This can be contrasted with single dipole solutions, which are typically based on one time point of phase-locked/evoked activity.

A third advantage is the increased spatial precision and region-specific interpretation of EEG activity. Although the spatial resolution of EEG and MEG is clearly inferior to MRI, the spatial resolution of scalp EEG can be improved through the application of beamforming, which can give results that are comparable to fMRI localization under ideal conditions of highly anatomically accurate forward models [60]. Although we did not use such anatomically precise forward models in the present study, future developments in forward model computation and electrode position measurements will further improve the spatial accuracy of beamforming reconstruction.

### Limitations

The main methodological limitation of this study is the decreased accuracy resulting from 64 electrodes and a lack of subject-specific anatomical forward models. This is sufficient for beamforming but results in greater uncertainty in source localization and decreased anatomical precision [61], [62], [63], and thus the spatial precision of the present study is considerably lower than that of functional MRI. In this sense, the purpose of using source reconstruction in the present study was not to answer the question “Where exactly in the brain does this occur?” but rather to utilize the mathematical framework of beamforming to construct adaptive spatial-temporal-frequency filters to highlight specific features of the data that might otherwise be difficult to detect at the level of the scalp. Improved spatial precision and accuracy could be achieved using high-density EEG (e.g., 128 or 256 electrodes) and subject-specific head models, and MEG recordings.

Because of the limited precision in anatomical localization, we focused on the inhibition of the task-irrelevant dimension (spatial location), rather than on the processing of the task-relevant dimension (stimulus color). Presumably, while spatial location information was suppressed, color processing was enhanced, as has been suggested for other stimulus features [64], [65]. It is unclear whether similar or different neurocognitive and oscillatory mechanisms are involved in activating task-relevant information compared to suppressing task-irrelevant information. Unfortunately, visual cortical areas involved in color processing (for example, V4/a) are difficult to localize precisely with 64-channel EEG because they are smaller and deeper sources (compared to MFC and parietal areas, which are larger and closer to the skull).

The time-lag in the parietal-frontal correlations is compelling and the temporal order of these neural events suggests a causal flow of information. However, causality per se is more difficult to establish in this dataset. Commonly used analysis methods for directed connectivity (e.g., Granger prediction and other related bivariate autoregressive modeling approaches) are not well suited for delays of hundreds of milliseconds because of the large number of free parameters that would be required, and have not yet been established for cross-frequency interactions. Combining the data analysis protocol of this study with stimulation of prefrontal or parietal areas via transcranial magnetic [29] or electrical stimulation would be an important follow-up to determine the causal contributions of prefrontal and parietal areas to spatial conflict processing and resolution.

### Conclusions

The application of beamforming to EEG data collected during a Simon task revealed the involvement of complementary cortical networks during the processing and resolution of action conflict. One network involves interactions between posterior MFC and ventrolateral PFC that operate in the theta band. It appears that when action conflicts are detected in MFC, additional activation in the IFG is recruited in order to support the inhibition and override of incorrect action. A second network involves interactions between stimulus-contralateral parietal cortex and ventrolateral PFC through cross-frequency coupling between parietal gamma and frontal theta. This connectivity may represent feed-forward activation from parietal cortex during congruent trials, which is suppressed during incongruent trials, and may reflect attempts to prevent the conflicting spatial location from capturing the corresponding hand action. A third network involves cross-frequency coupling between MFC theta and stimulus-contralateral parietal alpha. Early MFC theta was coupled to parietal alpha that occurred later, during the inter-trial-interval, rendering it less likely that this third network is involved directly in resolving action conflict.

## Methods

### Participants

Twenty subjects (8 male; aged 20–27) were recruited from the university undergraduate and graduate student population, and volunteered in exchange for course credit or money (€14). Subjects had normal or corrected-to-normal vision and no reported history of psychosis, brain disease, or psychiatric illness, and were self-reported right-handed. Subjects provided written consent prior to the start of the experiment, and all experimental procedures were approved by the local ethics committee at the University of Amsterdam.

### Task Design

Subjects performed a Simon task, in which a circle was presented to the left or the right visual hemifield (approximately 2.5° in diameter, 6.4° from fixation, at a distance of 90 cm). Subjects were instructed to respond, as quickly as possible, to the color of the stimulus (red and purple circle with left hand; blue and yellow circle with right hand) while ignoring its location (Figure 1a). Stimuli remained on screen for 200 ms, and subjects had up to 1000 ms thereafter to respond. A randomized inter-trial-interval of between 700 and 1200 ms separated trials. Conflict is induced when the response hand is opposite to the visual hemifield (e.g., red circle on the right side of the screen). Due to trial sequence effects (also called the “Gratton effect”) [43], [66], the “conflict effect” (the difference in behavior/brain activity between incongruent and congruent trials) depends on the congruence of the previous trial. Therefore, stimulus color and presentation side were pseudo-randomized such that there was an equal number and distribution of trial pairs: congruent-congruent, incongruent-congruent, congruent-incongruent, and incongruent-incongruent. These four trial types were pseudo-randomized, as was the order of color and stimulus presentation side, such that no consecutive trials contained identical stimulus parameters. Mapping two colors to each hand also avoids stimulus repetition effects [67], [68]. In the present study, we separately analyze trials following congruent trials to maximize the conflict effect [11]. We refer to trials in which the stimulus appeared in the “left hemifield” or the “right hemifield.” Note that the expected visual/spatial processing areas will be contralateral to stimulus hemifield. We did not perform any standard color vision tests, but we can infer that all subjects discriminated the four colors based on each subject’s task performance (overall accuracy and conflict effects).

Subjects first received instructions and one training block before the experiment began. All subjects correctly stated the instructions and color-response hand mapping. There were 1500 trials, with self-paced rest breaks occurred every 60 trials.

### EEG Acquisition and Preprocessing

EEG data were acquired at 2048 Hz from 64 BioSemi active electrodes (see www.biosemi.com for hardware details) placed according to the international 10–20 system. Electrode offsets were all kept within normal and acceptable ranges. Additional electrodes were placed to acquire horizontal eye movements and electromyographic signals from the thumbs. Reference electrodes were placed on both earlobes to serve as reference. Offline, EEG data were high-pass filtered at 0.5 Hz and then epoched from −1 to +1.5 seconds surrounding each trial. All trials were visually inspected and those containing EMG or other artifacts not related to blinks were manually removed. Independent components analysis was computed using the eeglab toolbox for Matlab [69] software, and components containing blink/oculomotor artifacts or other artifacts that could be clearly distinguished from brain-driven EEG signals were subtracted from the data.

The following trials were removed prior to analyses: Error trials (those in which subjects pressed the incorrect button or did not make a response) and trials following errors, trials with RTs shorter than 200 ms or longer than three standard deviations above each subject’s median RT, trials with partial errors (details below), trials with horizontal eye movements indicating saccades away from fixation, and the first trial following each rest period. Trials following incongruent trials were analyzed separately because behavioral results showed no conflict effect of trials following incongruent trials, as described above [43], [66]. Partial errors are when subjects made the correct response but twitched the muscle corresponding to the incorrect response, and elicit a qualitatively distinct pattern of brain dynamics compared to pure correct responses [70]. We identified a trial as containing a partial error if the Z-transformed derivative of the EMG signal exceeded one standard deviation between 200 ms post-stimulus onset and the actual button press, and if the peak of the partial error was more than two times the largest peak from −300 ms to stimulus onset (this eliminated trials with noisy EMG in the baseline period) [18].

Trials with horizontal eye movements that exceeded 5 standard deviations to either direction relative to pre-stimulus baseline activity were also removed from analyses (HEOG data were first low-pass filtered at 20 Hz). Although ICA may isolate artifacts resulting from horizontal eye movements, in our dataset few subjects had such a component, likely because subjects made relatively few saccades. Furthermore, eliminating those trials rather than correcting for the oculomotor artifact removed trials in which subjects broke fixation.

Because of trial sequence effects, the conflict effect is weak or absent (or sometimes reversed) on trials following incongruent trials [43], [66]. In the present data, it is apparent that the pattern of RTs and accuracy rates after incongruent trials was different from those following congruent trials (Figure 1b). Finally, all conditions were trial- and RT-matched, such that RT-matched trials were selected from the three (out of four) conditions to match the trial count of the condition with the fewest trials [18]. This procedure minimizes the possibility of condition differences in EEG activity resulting from differences in trial count or time-on-task (note that these trials were not excluded for behavioral analyses). However, this amounted to removing 30 trials on average per subject (thus, an average of 7.5 trials per condition), so it is unlikely to have altered any effects. Across subjects, an average of 47.66% (standard error: 3.5%) of trials were retained for analyses. There were on average 86.5 (stdev: 30.8) trials per condition for analyses. Although this may seem a strict set of criteria that removed many trials, we can be highly confident that the remaining trials are uncontaminated by EEG or methodological artifacts. The same trials were used for electrode-level analyses, source-space analyses, and behavior analyses (except that Figure 1b also shows results for trials following incongruent trials).

### EEG Electrode-based Analyses

Single-trial data were first decomposed into their time-frequency representation by multiplying the power spectrum of the EEG (obtained from the fast-Fourier-transform) by the power spectrum of complex Morlet wavelets (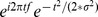, where *t* is time, *f* is frequency, which increased from 2 to 60 Hz in 30 logarithmically spaced steps, and 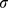 defines the width of each frequency band, set according to 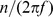 where *n* is the number of wavelet cycles, and increased from 3 to 13 in logarithmic steps), and then taking the inverse fast-Fourier-transform (i.e., frequency-domain convolution). From the resulting complex signal, an estimate of frequency-band-specific power at each time point was defined as the squared magnitude of the result of the convolution (real[z(t)]^2^+ imag[z(t)]^2^). Power was normalized using a decibel (dB) transform (dB power = 10*log10[power/baseline]), where the baseline activity was taken as the average power at each frequency band, averaged across conditions, from −300 to −100 ms pre-stimulus. DB conversion ensures that all frequencies, time points, electrodes, conditions, and subjects are in the same scale and thus comparable. Statistics were performed via t-tests across conditions at each electrode, using a significance threshold of p<0.00078125 (0.05/64, thus correcting for multiple comparisons across electrodes) and p<0.005 (uncorrected) (respectively, gray and white dots in topographical plots).

Although the focus of the current paper is on the source-space results, we report electrode-level analyses to replicate previously reported effects of conflict manipulations. These findings therefore help anchor the source-space results, and help link the source-space findings to other EEG studies that apply only electrode-level analyses. For this reason, we limited the electrode-level analyses to the same frequency bands used for the source-level analyses.

### EEG Source-based Analyses

Source reconstruction was performed via linear constrained minimum variance beamforming. These analyses were performed in Matlab using published equations [71], [72], and for completeness are described here. The idea of beamforming in the context of scalp electrophysiology is to construct a set of electrode weights in which the weighted sum of all electrodes estimates activity generated by a single location (voxel) in the brain. We estimated weights for 15,703 voxels, each 7 mm^3^, distributed throughout the cortex. The estimate of the source activity at each voxel v is a weighted sum of electrode data: **W_v_X**, where **W_v_** are the weights and **X** is the electrode data. **W_v_** is computed according to **(C^−1^L_v_)(L_v_^T^C^−1^L_v_)^−1^**, where **L_v_** is the leadfield (forward model), or the model of topographical activity given a source at voxel v and orientation that is active with unit gain, **C** is the sensor covariance, **^T^** indicates the matrix transpose, and**^−1^** indicates the inverse (here we used the pseudo-inverse, see next paragraph). Note that this formulation is similar to a weighted least squares fit, in which the best linear fit of the dependent variable (**C**, the data covariance) to the independent variable (**L**, the forward model) is computed. Each voxel has three orientations (one radial and two tangential); weights were adjusted towards the optimal power orientation using an approach outlined by Sekihara et al. [73], in which the optimal power value is computed based on the maximum eigenvalue of the source covariance matrix. This approach has been shown to optimize power estimates per voxel [73], and is particularly useful when the subject-specific brain anatomy and electrode positions are uncertain. The leadfield was computed using the openmeeg algorithm [74] using a standard MNI-space anatomical MRI, implemented in the Matlab toolbox BrainStorm [75]. Finally, band-specific power at each voxel is defined as **Y_v_ = W(CW)^T^**.

Because we used ICA to remove noise components from the data, the covariance matrices had reduced rank (generally, 62 rather than the full 64). We therefore used the pseudo-inverse in the equations above. Although this strategy, like noise regularization, results in some increased spatial blurring [76], it is a valid approach for beamforming using low-rank matrices [73]. Note that this did not affect the connectivity analyses (described in the next section), because we did not compute connectivity simultaneously in time or in frequency. Furthermore, condition comparisons will attenuate some spatial blurring.

To increase the signal-to-noise of the source reconstruction analyses, and also to reduce the total number of statistical comparisons, electrode covariance matrices were computed for time-frequency windows (“tiles”) [71]. We used 7 frequency bands (in Hz): 2–4, 4–8, 8–12, 12–30, 30–50, 50–80. Time windows were centered around time-points −200 to 1000 in 50 ms steps. The width of the time windows was related to the frequency band (in ms, with respect to frequency bands): 400, 300, 300, 200, 200, 200. Inhomogeneous time windowing is done because the robustness of covariance based on band-limited data is related to the number of cycles; thus, lower frequency bands should have longer time windows. To band-pass filter the data, we used Matlab’s zero-phase-lag *filtfilt* function. All trials from all conditions were pooled to compute the weights, and those weights were subsequently applied to condition-specific activity. This helped to further increase signal-to-noise ratio by maximizing the data used to compute weights.

Two features of the source power estimates require some transformation before interpretation and statistical testing. First, power values increase with distance from the scalp (though there are methods for scaling power by distance; [72]). Second, absolute power values are difficult to interpret, partly because of frequency power-law scaling (higher frequencies have lower power), and partly because the dynamics of interest are those specifically elicited by the task. Thus, power values were transformed into decibel, where the baseline period here was taken as the average power from tiles with center times from one half of the covariance window minus 100 ms pre-stimulus onset until 100 ms pre-stimulus onset (for example, for theta power, the baseline was −250 to −100 ms). This was done separately for each frequency band and at each voxel. Prior to statistics, data were spatially smoothed using a 20 mm^3^ Gaussian kernel (around 3 voxels) [77], which helps minimize inter-subject anatomical/functional variability. Statistical procedures are described below.

### Source Space Connectivity

We wished to examine “seeded” connectivity between specified frontal and parietal areas to the rest of the brain, without constraining the analyses to particular voxels or time-frequency tiles. We thus adapted an approach for using a time/frequency/brain region-of-interest as a seed, and correlating power in that seed, across trials, with power in the rest of time/frequency/brain space [46]. Seeds were selected based on a main effect of conflict (for MFC) or an interaction between conflict and stimulus hemifield (for parietal gamma). We extracted the single-trial gamma power from each time/frequency/brain region (defined as the supra-threshold voxels in the analysis of incongruent vs. congruent power), and correlated these cross-trial power fluctuations with power fluctuations in the rest of time-frequency-brain space. This resulted in a time/frequency/brain space of correlation coefficients corresponding to the extent to which cross-trial power fluctuations correlated with cross-trial fluctuations in the seed region.

This is an improvement over previous studies that use power correlations [41], [46] because our approach additionally allows the discovery of connectivity that occurs in different time-frequency windows (thus, cross-frequency and cross-time connectivity). Because significant correlations may arise to due fluctuations in global brain activation, we considered only regions in which the correlations were significantly different between incongruent and congruent trials. Statistical procedures were the same as for source power, described in the next section.

There are many different methods for assessing functional connectivity of M/EEG data, and few comprehensive formal comparisons among them. An advantage of the power-based connectivity analysis performed here (which is why we favored it in this study) is that it allows assessment of connectivity over time and frequency. This allowed us to identify, for example, that early parietal conflict-related gamma predicted later conflict-related frontal theta. In contrast, for example, most phase-based connectivity analyses assume simultaneous coupling at identical frequency bands.

### Source Space Statistics

Statistics on time-frequency changes in power were performed by map-wise t-tests, using a combination of masking, and pixel- and cluster-level thresholding, all based on non-parametric permutation testing [78], [79], [80]. The following procedure was done for each time-frequency tile. The first step was to mask voxels based on a significant main effect of task (all conditions pooled) vs. baseline, correcting for multiple comparisons across voxels. At each of 1000 iterations, data were multiplied by −1 for a random half of the subjects. From each iteration, the maximum t-value in the brain was stored, thus resulting in a distribution of maximum t-values expected under the null hypothesis. Voxels with a t-value greater than 95% of the null-distribution maximum t-values were considered significantly task-modulated, and subsequent analyses were performed only on these voxels. With this mask, we proceeded to test condition differences. Permutation testing was again performed by swapping condition labels across subjects. This time, at each of 1000 iterations the t-values at each voxel were stored, thus resulting in null-hypothesis t-value distributions at each voxel. Voxels were considered significant if their actual t-value was greater than 99% of the null t-values (thus, p<0.01). Finally, we applied cluster-correction. Clusters of voxels were considered significant if there were more voxels per cluster than expected under the null hypothesis at p<0.05. The following procedure was used to obtain a distribution of cluster sizes under the null hypothesis. At each iteration of permutation testing, the permuted brain map was thresholded at p<0.01, and the number of voxels in the largest supra-threshold cluster was stored. Because these brain maps reflected statistical results of random shuffling, any supra-threshold cluster would be considered a chance finding. After 1000 iterations, a distribution of maximum cluster sizes under the null hypothesis was created. Clusters in the real thresholded t-maps were discarded if they contained fewer voxels than 95% of null-distribution maximum cluster sizes. We believe that this three-pronged approach sufficiently minimized the possibility of observing spurious effects. Source-space results were thresholded such that only brain voxels that exceeded the significance threshold can be seen in the figures.

Results for all source analyses were viewed using nutmeg [81], a matlab toolbox for beamforming MEG data.

## References

[1] Ridderinkhof KR, van den Wildenberg WP, Segalowitz SJ, Carter CS (2004) Neurocognitive mechanisms of cognitive control: the role of prefrontal cortex in action selection, response inhibition, performance monitoring, and reward-based learning. Brain Cogn 56: 129–140. 15518930 10.1016/j.bandc.2004.09.016

[2] Botvinick MM, Braver TS, Barch DM, Carter CS, Cohen JD (2001) Conflict monitoring and cognitive control. Psychol Rev 108: 624–652. 11488380 10.1037/0033-295x.108.3.624

[3] Carter CS, van Veen V (2007) Anterior cingulate cortex and conflict detection: an update of theory and data. Cogn Affect Behav Neurosci 7: 367–379. 18189010 10.3758/cabn.7.4.367

[4] Hartwigsen G, Bestmann S, Ward NS, Woerbel S, Mastroeni C, et al (2012) Left Dorsal Premotor Cortex and Supramarginal Gyrus Complement Each Other during Rapid Action Reprogramming. J Neurosci 32: 16162–16171. 23152600 10.1523/JNEUROSCI.1010-12.2012 PMC3558742

[5] Kerns JG, Cohen JD, MacDonald AW 3rd, Cho RY, Stenger VA, et al (2004) Anterior cingulate conflict monitoring and adjustments in control. Science 303: 1023–1026. 14963333 10.1126/science.1089910

[6] Taylor PC, Nobre AC, Rushworth MF (2007) Subsecond changes in top down control exerted by human medial frontal cortex during conflict and action selection: a combined transcranial magnetic stimulation electroencephalography study. J Neurosci 27: 11343–11353. 17942729 10.1523/JNEUROSCI.2877-07.2007 PMC6673042

[7] Cavanagh JF, Cohen MX, Allen JJ (2009) Prelude to and resolution of an error: EEG phase synchrony reveals cognitive control dynamics during action monitoring. J Neurosci 29: 98–105. 19129388 10.1523/JNEUROSCI.4137-08.2009 PMC2742325

[8] Luu P, Tucker DM, Makeig S (2004) Frontal midline theta and the error-related negativity: neurophysiological mechanisms of action regulation. Clin Neurophysiol 115: 1821–1835. 15261861 10.1016/j.clinph.2004.03.031

[9] Trujillo LT, Allen JJ (2007) Theta EEG dynamics of the error-related negativity. Clin Neurophysiol 118: 645–668. 17223380 10.1016/j.clinph.2006.11.009

[10] Cavanagh JF, Frank MJ, Klein TJ, Allen JJ (2010) Frontal theta links prediction errors to behavioral adaptation in reinforcement learning. Neuroimage 49: 3198–3209. 19969093 10.1016/j.neuroimage.2009.11.080 PMC2818688

[11] Cohen MX, Cavanagh JF (2011) Single-trial regression elucidates the role of prefrontal theta oscillations in response conflict. Front Psychol 2: 30. 21713190 10.3389/fpsyg.2011.00030 PMC3111011

[12] Cohen MX, Ridderinkhof KR, Haupt S, Elger CE, Fell J (2008) Medial frontal cortex and response conflict: evidence from human intracranial EEG and medial frontal cortex lesion. Brain Res 1238: 127–142. 18760262 10.1016/j.brainres.2008.07.114

[13] Sauseng P, Klimesch W, Gruber WR, Hanslmayr S, Freunberger R, et al (2007) Are event-related potential components generated by phase resetting of brain oscillations? A critical discussion. Neuroscience 146: 1435–1444. 17459593 10.1016/j.neuroscience.2007.03.014

[14] Marco-Pallares J, Camara E, Munte TF, Rodriguez-Fornells A (2008) Neural mechanisms underlying adaptive actions after slips. J Cogn Neurosci 20: 1595–1610. 18345985 10.1162/jocn.2008.20117

[15] van de Vijver I, Ridderinkhof KR, Cohen MX (2011) Frontal oscillatory dynamics predict feedback learning and action adjustment. J Cogn Neurosci. 10.1162/jocn_a_00110 21812570

[16] Cohen MX (2011) Error-related medial frontal theta activity predicts cingulate-related structural connectivity. Neuroimage 55: 1373–1383. 21195774 10.1016/j.neuroimage.2010.12.072

[17] Cohen MX, van Gaal S, Ridderinkhof KR, Lamme VA (2009) Unconscious errors enhance prefrontal-occipital oscillatory synchrony. Front Hum Neurosci 3: 54. 19956401 10.3389/neuro.09.054.2009 PMC2786300

[18] Cohen MX, Van Gaal S (2012) Dynamic interactions between large-scale brain networks predict behavioral adaptation after perceptual errors. Cereb Cortex. 10.1093/cercor/bhs069 PMC3615344 22514250

[19] Nigbur R, Cohen MX, Ridderinkhof KR, Sturmer B (2012) Theta Dynamics Reveal Domain-specific Control over Stimulus and Response Conflict. J Cogn Neurosci 24: 1264–1274. 21861681 10.1162/jocn_a_00128

[20] Cohen MX, Bour L, Mantione M, Figee M, Vink M, et al (2012) Top-down-directed synchrony from medial frontal cortex to nucleus accumbens during reward anticipation. Hum Brain Mapp 33: 246–252. 21547982 10.1002/hbm.21195 PMC6870222

[21] Liu X, Banich MT, Jacobson BL, Tanabe JL (2004) Common and distinct neural substrates of attentional control in an integrated Simon and spatial Stroop task as assessed by event-related fMRI. Neuroimage 22: 1097–1106. 15219581 10.1016/j.neuroimage.2004.02.033

[22] Wittfoth M, Buck D, Fahle M, Herrmann M (2006) Comparison of two Simon tasks: neuronal correlates of conflict resolution based on coherent motion perception. Neuroimage 32: 921–929. 16677831 10.1016/j.neuroimage.2006.03.034

[23] Wittfoth M, Kustermann E, Fahle M, Herrmann M (2008) The influence of response conflict on error processing: evidence from event-related fMRI. Brain Res 1194: 118–129. 18177843 10.1016/j.brainres.2007.11.067

[24] Walsh BJ, Buonocore MH, Carter CS, Mangun GR (2011) Integrating conflict detection and attentional control mechanisms. J Cogn Neurosci 23: 2211–2221. 21126158 10.1162/jocn.2010.21595 PMC3142580

[25] Sturmer B, Leuthold H (2003) Control over response priming in visuomotor processing: a lateralized event-related potential study. Exp Brain Res 153: 35–44. 12955386 10.1007/s00221-003-1579-1

[26] Appelbaum LG, Smith DV, Boehler CN, Chen WD, Woldorff MG (2011) Rapid modulation of sensory processing induced by stimulus conflict. J Cogn Neurosci 23: 2620–2628. 20849233 10.1162/jocn.2010.21575 PMC3096678

[27] Jin Y, Olk B, Hilgetag CC (2010) Contributions of human parietal and frontal cortices to attentional control during conflict resolution: a 1-Hz offline rTMS study. Exp Brain Res 205: 131–138. 20617309 10.1007/s00221-010-2336-x

[28] Rusconi E, Turatto M, Umilta C (2007) Two orienting mechanisms in posterior parietal lobule: an rTMS study of the Simon and SNARC effects. Cogn Neuropsychol 24: 373–392. 18416497 10.1080/02643290701309425

[29] Schiff S, Bardi L, Basso D, Mapelli D (2011) Timing spatial conflict within the parietal cortex: a TMS study. J Cogn Neurosci 23: 3998–4007. 21671746 10.1162/jocn_a_00080

[30] Bardi L, Kanai R, Mapelli D, Walsh V (2012) TMS of the FEF Interferes with Spatial Conflict. J Cogn Neurosci. 10.1162/jocn_a_00223 22401287

[31] Milner AD, Goodale MA (1995) The visual brain in action. Oxford: Oxford University Press.

[32] Behrmann M, Geng JJ, Shomstein S (2004) Parietal cortex and attention. Curr Opin Neurobiol 14: 212–217. 15082327 10.1016/j.conb.2004.03.012

[33] Rushworth MF, Ellison A, Walsh V (2001) Complementary localization and lateralization of orienting and motor attention. Nat Neurosci 4: 656–661. 11369949 10.1038/88492

[34] Worden MS, Foxe JJ, Wang N, Simpson GV (2000) Anticipatory biasing of visuospatial attention indexed by retinotopically specific alpha-band electroencephalography increases over occipital cortex. J Neurosci 20: RC63. 10704517 10.1523/JNEUROSCI.20-06-j0002.2000 PMC6772495

[35] Van Der Werf J, Buchholz VN, Jensen O, Medendorp WP (2012) Reorganization of Oscillatory Activity in Human Parietal Cortex during Spatial Updating. Cereb Cortex. 10.1093/cercor/bhr387 22414770

[36] Siegel M, Donner TH, Oostenveld R, Fries P, Engel AK (2008) Neuronal synchronization along the dorsal visual pathway reflects the focus of spatial attention. Neuron 60: 709–719. 19038226 10.1016/j.neuron.2008.09.010

[37] Palva S, Kulashekhar S, Hamalainen M, Palva JM (2011) Localization of cortical phase and amplitude dynamics during visual working memory encoding and retention. J Neurosci 31: 5013–5025. 21451039 10.1523/JNEUROSCI.5592-10.2011 PMC3083635

[38] Palva S, Monto S, Palva JM (2010) Graph properties of synchronized cortical networks during visual working memory maintenance. Neuroimage 49: 3257–3268. 19932756 10.1016/j.neuroimage.2009.11.031

[39] Phillips S, Takeda Y (2009) Greater frontal-parietal synchrony at low gamma-band frequencies for inefficient than efficient visual search in human EEG. Int J Psychophysiol 73: 350–354. 19481120 10.1016/j.ijpsycho.2009.05.011

[40] Buschman TJ, Miller EK (2007) Top-down versus bottom-up control of attention in the prefrontal and posterior parietal cortices. Science 315: 1860–1862. 17395832 10.1126/science.1138071

[41] Mazaheri A, Nieuwenhuis IL, van Dijk H, Jensen O (2009) Prestimulus alpha and mu activity predicts failure to inhibit motor responses. Hum Brain Mapp 30: 1791–1800. 19308934 10.1002/hbm.20763 PMC6870709

[42] Mazaheri A, Coffey-Corina S, Mangun GR, Bekker EM, Berry AS, et al (2010) Functional disconnection of frontal cortex and visual cortex in attention-deficit/hyperactivity disorder. Biol Psychiatry 67: 617–623. 20060100 10.1016/j.biopsych.2009.11.022

[43] Egner T (2007) Congruency sequence effects and cognitive control. Cogn Affect Behav Neurosci 7: 380–390. 18189011 10.3758/cabn.7.4.380

[44] Cavanagh JF, Zambrano-Vazquez L, Allen JJ (2012) Theta lingua franca: a common mid-frontal substrate for action monitoring processes. Psychophysiology 49: 220–238. 22091878 10.1111/j.1469-8986.2011.01293.x PMC3262926

[45] Nigbur R, Ivanova G, Sturmer B (2011) Theta power as a marker for cognitive interference. Clin Neurophysiol 122: 2185–2194. 21550845 10.1016/j.clinph.2011.03.030

[46] Yeung N, Bogacz R, Holroyd CB, Nieuwenhuis S, Cohen JD (2007) Theta phase resetting and the error-related negativity. Psychophysiology 44: 39–49. 17241139 10.1111/j.1469-8986.2006.00482.x

[47] Agam Y, Hamalainen MS, Lee AK, Dyckman KA, Friedman JS, et al (2011) Multimodal neuroimaging dissociates hemodynamic and electrophysiological correlates of error processing. Proc Natl Acad Sci U S A 108: 17556–17561. 21969565 10.1073/pnas.1103475108 PMC3198335

[48] Ridderinkhof KR, Forstmann BU, Wylie SA, Burle B, Van den Wildenberg W (2011) Neurocognitive mechanisms of action control: Resisting the call of the Sirens. Wylie Interdisciplinary Reviews: Cognitive Science 2: 174–192. 26302009 10.1002/wcs.99

[49] Donner TH, Siegel M (2011) A framework for local cortical oscillation patterns. Trends Cogn Sci 15: 191–199. 21481630 10.1016/j.tics.2011.03.007

[50] Jensen O, Bonnefond M, VanRullen R (2012) An oscillatory mechanism for prioritizing salient unattended stimuli. Trends Cogn Sci 16: 200–206. 22436764 10.1016/j.tics.2012.03.002

[51] Mathewson KE, Lleras A, Beck DM, Fabiani M, Ro T, et al (2011) Pulsed out of awareness: EEG alpha oscillations represent a pulsed-inhibition of ongoing cortical processing. Front Psychol 2: 99. 21779257 10.3389/fpsyg.2011.00099 PMC3132674

[52] Compton RJ, Arnstein D, Freedman G, Dainer-Best J, Liss A (2011) Cognitive control in the intertrial interval: evidence from EEG alpha power. Psychophysiology 48: 583–590. 20840195 10.1111/j.1469-8986.2010.01124.x

[53] Compton RJ, Huber E, Levinson AR, Zheutlin A (2012) Is “conflict adaptation” driven by conflict? Behavioral and EEG evidence for the underappreciated role of congruent trials. Psychophysiology 49: 583–589. 22332754 10.1111/j.1469-8986.2012.01354.x

[54] Ruiz MH, Strubing F, Jabusch HC, Altenmuller E (2011) EEG oscillatory patterns are associated with error prediction during music performance and are altered in musician’s dystonia. Neuroimage 55: 1791–1803. 21195188 10.1016/j.neuroimage.2010.12.050

[55] Levy BJ, Wagner AD (2011) Cognitive control and right ventrolateral prefrontal cortex: reflexive reorienting, motor inhibition, and action updating. Ann N Y Acad Sci 1224: 40–62. 21486295 10.1111/j.1749-6632.2011.05958.x PMC3079823

[56] Canolty RT, Knight RT (2010) The functional role of cross-frequency coupling. Trends Cogn Sci 14: 506–515. 20932795 10.1016/j.tics.2010.09.001 PMC3359652

[57] van der Meij R, Kahana M, Maris E (2012) Phase-amplitude coupling in human electrocorticography is spatially distributed and phase diverse. J Neurosci 32: 111–123. 22219274 10.1523/JNEUROSCI.4816-11.2012 PMC6621324

[58] Palva S, Palva JM (2007) New vistas for alpha-frequency band oscillations. Trends Neurosci 30: 150–158. 17307258 10.1016/j.tins.2007.02.001

[59] Rohenkohl G, Nobre AC (2011) alpha oscillations related to anticipatory attention follow temporal expectations. J Neurosci 31: 14076–14084. 21976492 10.1523/JNEUROSCI.3387-11.2011 PMC4235253

[60] Singh KD, Barnes GR, Hillebrand A, Forde EM, Williams AL (2002) Task-related changes in cortical synchronization are spatially coincident with the hemodynamic response. Neuroimage 16: 103–114. 11969322 10.1006/nimg.2001.1050

[61] Ding J, Sperling G, Srinivasan R (2006) Attentional modulation of SSVEP power depends on the network tagged by the flicker frequency. Cereb Cortex 16: 1016–1029. 16221931 10.1093/cercor/bhj044 PMC1880883

[62] Michel CM, Murray MM, Lantz G, Gonzalez S, Spinelli L, et al (2004) EEG source imaging. Clin Neurophysiol 115: 2195–2222. 15351361 10.1016/j.clinph.2004.06.001

[63] Green JJ, McDonald JJ (2009) A Practical Guide to Beamformer Source Reconstruction for EEG. In: Handy TC, editor. Brain Signal Analysis: Advances in neuroelectric and neuromagnetic methods. Cambridge, MA: MIT Press. 79–98.

[64] Egner T, Hirsch J (2005) Cognitive control mechanisms resolve conflict through cortical amplification of task-relevant information. Nat Neurosci 8: 1784–1790. 16286928 10.1038/nn1594

[65] King JA, Korb FM, von Cramon DY, Ullsperger M (2010) Post-error behavioral adjustments are facilitated by activation and suppression of task-relevant and task-irrelevant information processing. J Neurosci 30: 12759–12769. 20861380 10.1523/JNEUROSCI.3274-10.2010 PMC6633589

[66] Gratton G, Coles MG, Donchin E (1992) Optimizing the use of information: strategic control of activation of responses. J Exp Psychol Gen 121: 480–506. 1431740 10.1037//0096-3445.121.4.480

[67] Wuhr P, Ansorge U (2005) Exploring trial-by-trial modulations of the Simon effect. Q J Exp Psychol A 58: 705–731. 16104103 10.1080/02724980443000269

[68] Cho RY, Orr JM, Cohen JD, Carter CS (2009) Generalized signaling for control: evidence from postconflict and posterror performance adjustments. J Exp Psychol Hum Percept Perform 35: 1161–1177. 19653756 10.1037/a0014491 PMC2881467

[69] Delorme A, Makeig S (2004) EEGLAB: an open source toolbox for analysis of single-trial EEG dynamics including independent component analysis. J Neurosci Methods 134: 9–21. 15102499 10.1016/j.jneumeth.2003.10.009

[70] Holroyd CB, Coles MG (2002) The neural basis of human error processing: reinforcement learning, dopamine, and the error-related negativity. Psychol Rev 109: 679–709. 12374324 10.1037/0033-295X.109.4.679

[71] Dalal SS, Guggisberg AG, Edwards E, Sekihara K, Findlay AM, et al (2008) Five-dimensional neuroimaging: localization of the time-frequency dynamics of cortical activity. Neuroimage 40: 1686–1700. 18356081 10.1016/j.neuroimage.2008.01.023 PMC2426929

[72] Van Veen BD, van Drongelen W, Yuchtman M, Suzuki A (1997) Localization of brain electrical activity via linearly constrained minimum variance spatial filtering. IEEE Trans Biomed Eng 44: 867–880. 9282479 10.1109/10.623056

[73] Sekihara K, Nagarajan SS (2008) Adaptive Spatial Filters for Electromagnetic Brain Imaging. London: Springer London, Limited.

[74] Gramfort A, Papadopoulo T, Olivi E, Clerc M (2011) Forward field computation with OpenMEEG. Comput Intell Neurosci 2011: 923703. 21437231 10.1155/2011/923703 PMC3061324

[75] Tadel F, Baillet S, Mosher JC, Pantazis D, Leahy RM (2011) Brainstorm: a user-friendly application for MEG/EEG analysis. Comput Intell Neurosci 2011: 879716. 21584256 10.1155/2011/879716 PMC3090754

[76] Woolrich M, Hunt L, Groves A, Barnes G (2011) MEG beamforming using Bayesian PCA for adaptive data covariance matrix regularization. Neuroimage 57: 1466–1479. 21620977 10.1016/j.neuroimage.2011.04.041 PMC4894461

[77] Barnes GR, Hillebrand A, Fawcett IP, Singh KD (2004) Realistic spatial sampling for MEG beamformer images. Hum Brain Mapp 23: 120–127. 15340934 10.1002/hbm.20047 PMC6872013

[78] Maris E, Oostenveld R (2007) Nonparametric statistical testing of EEG- and MEG-data. J Neurosci Methods 164: 177–190. 17517438 10.1016/j.jneumeth.2007.03.024

[79] Nichols TE, Holmes AP (2002) Nonparametric permutation tests for functional neuroimaging: a primer with examples. Hum Brain Mapp 15: 1–25. 11747097 10.1002/hbm.1058 PMC6871862

[80] Singh KD, Barnes GR, Hillebrand A (2003) Group imaging of task-related changes in cortical synchronisation using nonparametric permutation testing. Neuroimage 19: 1589–1601. 12948714 10.1016/s1053-8119(03)00249-0

[81] Dalal SS, Zumer JM, Agrawal V, Hild KE, Sekihara K, et al (2004) NUTMEG: a neuromagnetic source reconstruction toolbox. Neurol Clin Neurophysiol 2004: 52. 16012626 PMC1360185

